# Sleep trajectories and frequency of non-suicidal self-injury in adolescents: a person-oriented perspective over two years

**DOI:** 10.1038/s41598-025-85779-5

**Published:** 2025-01-11

**Authors:** S. V. Bauducco, L. Tilton-Weaver, M. Gradisar, M. Hysing, D. Latina

**Affiliations:** 1 Department of Psychology, Örebro University, Örebro, Sweden; 2 Department of Psychology, Flinders University, Adelaide, Australia; 3 WINK Sleep, Adelaide, Australia; 4 Sleep Cycle, Gothenburg, Sweden; 5 Department of Psychosocial Science, University of Bergen, Bergen, Norway; 6 Department of Child and Adolescents Psychiatry and Psychotherapy, University of Ulm, Ulm, Germany; 7 Center for Health and Medical Psychology, Örebro, Sweden

**Keywords:** Self harm, Sleep disturbance, Teenagers, Person-oriented analyses, Risk factors, Paediatric research

## Abstract

Adolescent sleep quality and quantity is commonly linked to worse emotion regulation. One maladaptive emotion regulation strategy that is on the rise is non-suicidal self-injury (NSSI), which includes burning, hitting, or scratching one’s own body tissue without suicidal intent. The aim of this study was to explore the frequency of NSSI among different longitudinal trajectories of insomnia symptoms and short sleep duration to identify at-risk adolescents. We used questionnaire data collected annually (3 time points over 2 years) from a sample of Swedish adolescents (N = 1,294; M_age_ = 13.2 [range: 12–15 years], SD = 0.4; 46.8% girls). Adolescents answered questions about their sleep duration, symptoms of insomnia, NSSI, depressive symptoms, and demographics. Adolescents who reported persistent or increasing sleep problems over time also reported more NSSI. A notable pattern was that adolescents whose insomnia symptoms were high and increasing reported the highest frequency of NSSI, also compared to adolescents who started at the same high level of insomnia symptoms but improved over time. Therefore, measuring NSSI may help identify a risk-group for persistent sleep problems and self-injury. Because sleep disturbances, especially insomnia, and NSSI go hand-in-hand for most adolescents, sleep interventions would benefit the treatment and prevention of self-injury.

## Introduction

Increasing attention has turned to the declining trends in sleep duration and sleep quality over the last decades^1^ and the close association between sleep and mental health in young people^2^. Numerous studies show that adolescents who sleep poorly also have difficulties regulating their emotions^3^, and one maladaptive emotion regulation strategy that is on the rise is non-suicidal self-injury (NSSI)^4,5^. For example, prevalence rates of lifetime NSSI increased from 2.4% in 2000 to 4.6% in 2014, especially in girls aged 16–24 years (from 6.5% in 2000 to 17.9% in 2014)^5^. It is therefore crucial that research identifies *who* is at risk for NSSI to inform preventive efforts. In the present study, we aim to investigate differences in NSSI frequency across adolescents’ longitudinal trajectories of insomnia symptoms and short sleep duration.

NSSI includes actions such as burning, hitting, or scratching the surface of one’s own body, and is performed in the absence of suicidal ideations^6^. Although NSSI serves several functions, affect regulation seems to be the most common, as NSSI helps adolescents to deal with emotions experienced as overwhelming or difficult to handle^7^. On average 17% of adolescents worldwide have self-injured at least once in their lifetime^8^, with Sweden reaching rates of up to 42%^9^. The typical age of onset is between 13 and 16 years^10^. Given that NSSI is a strong risk factor for suicidal ideations and attempts^11^, this phenomenon has become a growing public health concern^12^.

Sleep problems represent another health concern during adolescence, the most common include symptoms of insomnia and short sleep duration^13^. Among symptoms of insomnia, difficulties falling asleep are common because of a delayed sleep phase combined with early school times^14^. Between 4 and 39% of adolescents report symptoms of insomnia^15^. Aside from insomnia symptoms, millions of adolescents around the world suffer from insufficient sleep, defined as less than 7 h sleep/night for this age group^16^. For example, between 14 and 68% of adolescents from 24 European and North American countries reported insufficient sleep^17^. Of relevance to the present study, adolescents who report worse sleep quality and quantity consistently show poor emotion regulation^3^.

Sleep problems—including insomnia and short sleep duration—are associated with NSSI in community samples of adolescents^18–20^. Compared to good-sleeping adolescents, those reporting a different range of sleep difficulties (e.g., insomnia, short sleep duration, non-restorative sleep, frequent nightmares) are at higher risk of engaging in NSSI^21^. In addition, adolescents with sleep difficulties are at higher risk to injure themselves^19,22^. However, these findings primarily come from cross-sectional data, which does not allow us to draw temporal conclusions about this association. Only a limited number of studies have used a longitudinal design with two or three waves of data to explore this topic.

These longitudinal studies have reported a higher likelihood of recent or repetitive NSSI/self-harm for those adolescents reporting poor sleep^23^, insomnia symptoms^24^ or difficulties initiating sleep^25^. The majority controlled for depressive symptoms (except^23^), a well-known correlate of both sleep problems^3^ and NSSI^26^. Additionally, one study showed a bidirectional association between insomnia symptoms and NSSI^27^. However, these longitudinal studies used *variable-oriented approaches*, which fail to provide information on the course of these behaviors in different groups of adolescents. In this respect, recent work provides evidence supporting the hypothesis that distinct insomnia and sleep duration trajectories can be identified in adolescents^28^. For instance, one study showed that adolescents belonging to a consistently low and rapidly decreasing time in bed trajectory were more likely to engage in NSSI^29^. However, time in bed is not informative of adolescents’ sleep quantity, as for example, many perform activities when awake in bed (e.g., technology use). Nor does this approach inform about the quality of sleep.

In the present study, we aim to expand our knowledge of the sleep trajectories of at-risk adolescents, by examining NSSI levels in both sleep duration and insomnia symptom trajectories from early- to mid-adolescence. Based on previous findings^24,29^ we hypothesized that adolescents reporting insomnia symptoms and short sleep duration, as well as increasing insomnia symptoms and decreasing sleep duration, would show an increased risk of NSSI. To our knowledge, this is the first study of its kind that examines the occurrence of NSSI in groups of adolescents who follow different trajectories of both insomnia and sleep duration over a 2-year span (three time points).

## Results

### Adolescents’ trajectories of insomnia symptoms and sleep duration

Means and standard deviations of the study variables (i.e., insomnia symptoms, sleep duration, NSSI, demographic variables, and baseline depression) are reported in Table 1, for the whole sample, and boys and girls separately.

**Table 1:** Means and standard deviations of sleep duration, insomnia, NSSI and control variables (SES, immigrant background, depressive symptoms) in the whole sample and boys/girls separately.

Variables	Total sample (N = 1295) M(SD)	Boys (N = 689) M(SD)	Girls (N = 606) M(SD)
Sleep			
Sleep duration T1	8:07 h (1:07)	8:22 h (1:00)***	7:51 h (1:12) ***
Sleep duration T2	7:57 h (1:04)	8:11 h (0:59) ***	7:40 h (1:05) ***
Sleep duration T3	7:43 h (1:07)	7:52 (1:05) ***	7:32 h (1:08) ***
Insomnia symptoms T1	5.05 (4.74)	4.37 (4.23) ***	5.86 (5.12) ***
Insomnia symptoms T2	5.29 (4.85)	4.26 (4.22) ***	6.50 (5.26) ***
Insomnia symptoms T3	6.25 (5.61)	5.13 (5.05) ***	7.47 (5.89) ***
Non-suicidal self-injury			
NSSI T1	0.12 (0.42)	0.10 (0.40)*	0.15 (0.45)*
NSSI T2	0.15 (0.48)	0.06 (0.24) ***	0.26 (0.64) ***
NSSI T3	0.20 (0.62)	0.10 (0.40) ***	0.32 (0.79) ***
Control variables			
SES	6.70 (1.61)	6.68 (1.58)	6.77 (1.60)
Immigrant background	22%	21.2%	22.9%
Depressive symptoms T1	11.62 (10.53)	8.37 (8.18) ***	15.32 (11.63) ***

Insomnia symptoms scores range (0–28), Non-suicidal self-injury (NSSI) scores range (0–5), SES range (0–9), Depressive symptoms scores range (0–60). Significant differences between boys and girls are marked with asterisks (* = 0.05, *** < 0.001).

**Fig. 1:**
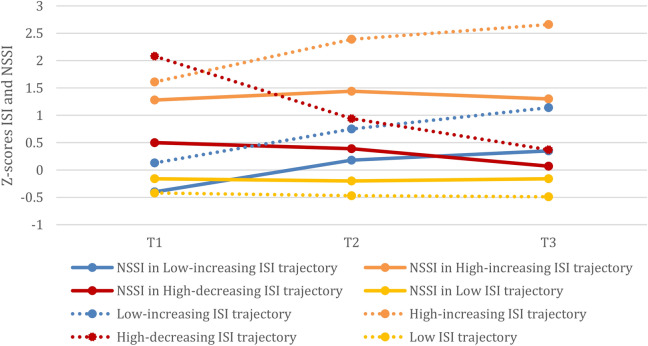
NSSI frequency in insomnia symptom trajectories at T1-T2-T3 (with insomnia symptom trajectories for reference). *Note.* Z-scores were used to show NSSI and ISI scores in the same figure. Lines of the same color refer to the same insomnia trajectory; dotted lines represent the insomnia symptom levels and solid lines represent NSSI levels in each insomnia trajectory, not controlling for confounders. *NSSI* non-suicidal self-injury, *ISI* Insomnia Severity Index. This figure was adapted from the figure published in Bauducco et al.^28^ under the CC BY 4.0 license.

**Fig. 2:**
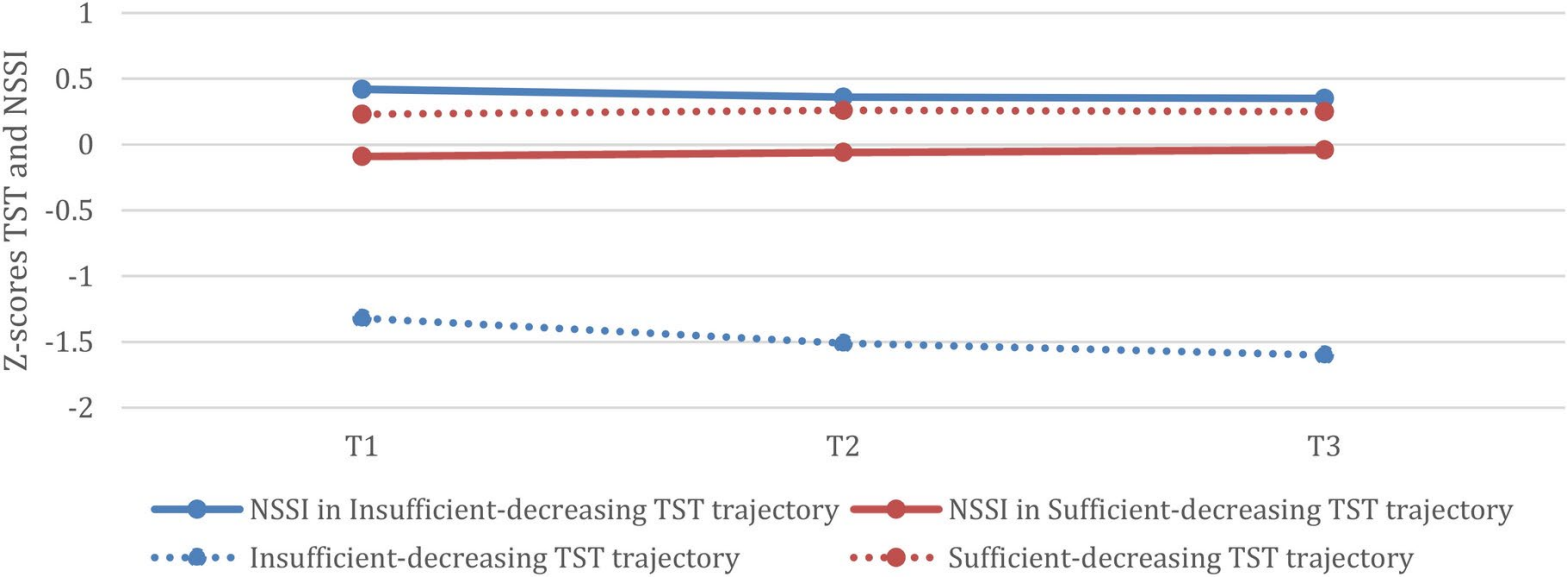
NSSI frequency in sleep duration trajectories at T1-T2-T3 (with Sleep duration trajectories for reference). *Note.* Z-scores were used to show NSSI and sleep duration scores in the same figure. Lines of the same color refer to the same sleep duration trajectory; dotted lines represent average sleep duration and solid lines represent NSSI scores in each sleep duration trajectory, without controlling for confounders. *NSSI* non-suicidal self-injury, *TST* total sleep time. This figure was adapted from the figure published in Bauducco et al.^28^ under the CC BY 4.0 license.

We have previously reported on the insomnia and sleep duration trajectories^28^. Overall, there was an increase in insomnia symptoms and a decrease in sleep duration. We identified 4 insomnia symptom trajectories and 2 sleep duration trajectories (see Figs. 1, 2 and^28^). The insomnia trajectories included a *low-increasing* trajectory (17.2% of the sample) reporting low levels of insomnia symptoms at T1 and sub-threshold symptoms at T3; a *high-increasing* trajectory (4.6% of the sample) reporting sub-threshold symptoms of insomnia at T1 and increased to the upper limit of moderate insomnia at T3; a *high-decreasing* trajectory (9% of the sample) reporting sub-threshold symptoms of insomnia at T1 and a substantial decrease by T3; and a *low insomnia* trajectory (69.2% of the sample) reporting low levels of insomnia symptoms at T1 and a slight increase over time. The sleep duration trajectories included a *sufficient-decreasing* trajectory (84.9% of the sample) showing a significant decline in sleep duration from an average of 8.4 to 8.0 h of sleep/weeknight; and an *insufficient-decreasing* trajectory (15.1% of the sample) showing a significant decline from 6.7 h/night at T1 to 6.1 h/night at T3.

### Adolescents’ trajectories of insomnia symptoms: differences and similarities in NSSI

We examined whether adolescents in different insomnia symptom trajectories differed in NSSI frequency, controlling for sex, SES, immigrant background, and baseline depressive symptoms (Table 2).

**Table 2:** Differences and similarities in demographics and NSSI at T1, T2, and T3 by insomnia trajectories.

	Low insomnia M (SD)	High increasing M (SD)	High decreasing M (SD)	Low increasing M (SD)	F(df)	*p*	Partial η^2^
NSSI T1	0.09^a^ (0.01)	0.51^b^ (0.05)	0.22^c^ (.04)	0.08^a^ (0.03)	21.38 (3,1287)	< 0.001	0.05
NSSI T2	0.10^a^ (0.02)	0.66^b^ (0.06)	0.24^c^ (.04)	0.20^c^ (0.03)	27.84 (3,1287)	< 0.001	0.06
NSSI T3	0.13^a^ (0.02)	0.80^b^ (0.08)	0.23^ac^ (0.06)	0.35^c^ (0.04)	25.83 (3,1287)	< 0.001	0.06

Non-suicidal self-injury (NSSI) response range (0–5), adjusted for control variables (SES, immigrant background, sex, and baseline depression).

Adolescents in the *high-increasing* insomnia symptom group reported the highest levels of NSSI (average number of NSSI events) at all three time points (Mean = 0.51; 0.66; 0.80, respectively), compared to the other groups. In contrast, adolescents in the *low insomnia* group consistently reported the lowest levels of NSSI (Mean = 0.09; 0.10; 0.13, respectively). Adolescents in the two divergent trajectories of insomnia—*high-decreasing* and *low-increasing* symptoms—showed a reversed pattern of NSSI frequency. At T1, adolescents in the *low-increasing* insomnia group did not differ from the *low insomnia* group (Mean = 0.08 vs 0.09), while at T3 they showed significantly higher levels than the *low insomnia* group (Mean = 0.35 vs 0.13). Moreover, adolescents in the *high-decreasing* group reported higher rates of NSSI at T1, compared to both *low insomnia* and *low-increasing* groups (Mean = 0.22 vs 0.08/0.09), at T3 NSSI levels no longer differed from the *low insomnia* group (Mean = 0.23 vs 0.13). However, NSSI frequency was not significantly different between the *high-decreasing* and *low-increasing* group at T3 (Mean = 0.23 vs 0.35). Compared to all other insomnia groups, adolescents in the *high-increasing* group reported significantly higher rates of NSSI at baseline (see Fig. 1).

### Adolescents’ trajectories of sleep duration: differences and similarities in NSSI

We investigated whether adolescents in different sleep duration trajectories differed in NSSI frequency, controlling for sex, SES, immigrant background, and baseline depressive symptoms (Table 3).

**Table 3:** Differences and similarities in demographics and NSSI at T1, T2, and T3 by sleep duration trajectories.

	Insufficient-decreasing M (SD)	Sufficient-decreasing M (SD)	F(df)	*p*	Partial η^2^
NSSI T1	0.24 (0.03)	0.10 (0.01)	20.51 (1,1289)	< 0.001	0.02
NSSI T2	0.23 (0.03)	0.14 (0.01)	6.58 (1,1289)	= 0.01	0.01
NSSI T3	0.39 (0.04)	0.17 (0.02)	21.36 (1,1289)	< 0.001	0.02

Non-suicidal self-injury (NSSI) response range (0–5), adjusted for control variables (SES, immigrant background, sex, and baseline depression).

NSSI frequency (average number of NSSI events) was consistently higher at all three time points among adolescents in the *insufficient-decreasing* (Mean = 0.24; 0.23; 0.39) sleep duration trajectory as compared to the *adequate-decreasing* (Mean = 0.10; 0.14; 0.17; see Fig. 2).

## Discussion

Our aim was to examine how temporal patterns of sleep problems over three years were related to non-suicidal self-injury. Our results suggest several clear trends.

In our study, self-injury changed hand-in-hand with different types of sleep problems for most adolescents. An important question that arises is whether one problem induces the other. In a study examining the temporal patterning of sleep and self-injury^27^, self-injury more consistently predicted changes in insomnia. However, from middle-to-late adolescence, insomnia and self-injury predicted changes in each other. By contrast, our results are from a person-centered analysis, compared to the variable-centered approach of lag models. That is, our focus was on *individual-level change* of sleep problems, rather than mean-level or rank-order change, and how NSSI risk differed across adolescents’ sleep trajectories. Our results suggest that for many, sleep difficulties and self-injury rise and abate together. Where sleep problems were persistent, there was more self-injury; where sleep problems were low, so was self-injury. If the two problems co-occur, developmental frameworks need to be adjusted, to address what may be transdiagnostic processes. Of course, future studies using shorter lags between data collections might show temporal patterns unseen in the present study. For example, a recent study using a daily ecological momentary assessment protocol with undergraduate students found that sleep irregularity was associated with daily NSSI urge intensity^30^.

However, our use of a person-centered approach to sleep problems revealed a pattern that would not have been seen with variable-centered approaches. The subgroup whose insomnia started high and increased over time had significantly higher levels of self-injury that remained high over time, compared to the other groups. Specifically, compared to adolescents in the *high-decreasing* trajectory, NSSI frequency was markedly higher in the *high-increasing* insomnia group at T1, despite having similar levels of insomnia symptoms at that same timepoint (see Fig. 1). These adolescents may represent the “psychopathology” pathway to NSSI, characterized by early and persistent emotional problems, that was identified in a recent longitudinal study^24^. Our results suggest that their onset of self-injury is not strongly tied to insomnia but has a different source, yet the two are highly comorbid over time.

Another important result is that NSSI seems to be linked to sleep disturbances independent of sex. Nevertheless, girls were more likely to be classified in the trajectories with higher levels of insomnia and shorter sleep duration^28^. Therefore, if sleep problems and self-injury tend to co-occur, this problem may be particularly critical for girls. Theoretically and empirically, there are other internalizing problems, such as symptoms of depression and anxiety, that have higher prevalence in girl, and are related to experiencing sleep problems, and self-injuring^15^. Our results could mean that girls are particularly vulnerable given the higher rate of both sleep problems and NSSI for girls, although the association is similar across sexes.

Our results suggest young adolescents with insomnia symptoms and/or insufficient sleep are at greater risk for elevated levels of NSSI. The co-occurrence of self-injury and sleep problems indicates the need to address insomnia and/or insufficient sleep in the prevention and treatment of both mental health issues. The inclusion of sleep interventions has been indicated as an important and integral part of interventions in relation to self-harm^31^. In addition, adolescents might be more prone to talk about sleep problems than NSSI, which may be perceived as more stigmatizing. Thus, the fields of sleep medicine and self-injury would do well to collaborate and form effective interventions with the ultimate aim of reducing non-suicidal self-injury in adolescents.

The strengths of the current study include longitudinal data, with a relatively large sample of adolescents during an important developmental period (i.e., from early adolescence). This time frame is likely to capture the onset of NSSI for most adolescents^10^ in relation to the sleep trajectories studied. In addition, NSSI was measured with multiple types of self-injury. Although cutting is the most common method used by individuals with a history of self-injury^5,32^, it is not the only one, and some adolescents do not predilect exclusively one form of self-injury over the others^32^. Our measure allowed us to assess several forms of self-injury, and thus provides a more accurate picture of this conduct in our community sample of adolescents. Finally, the person-oriented approach revealed an important risk group with persistent insomnia and the highest frequency of NSSI that would not be seen using variable-oriented analyses.

Despite the strengths of this study, the results should be interpreted considering the context of some limitations. All measures used were self-reported and could be affected by common method variance. More comprehensive measurement of both sleep and NSSI could have strengthened the study, for example by including a validated objective measure of sleep duration (e.g., polysomnography), and a clinical interview for insomnia. On the other hand, questionnaires are practical when including a large number of participants^33^. Moreover, we measured for key demographic characteristics and depressive symptoms, which is a strength of the present study, but there could be unmeasured confounds that may have affected the results. Although we explored both sleep duration and quality, which are the most common sleep problems during adolescence, future studies should also include other aspects of sleep that are emerging as closely linked to NSSI, such as sleep irregularity^30^. Further, the analyses adopted in this study cannot clarify why these associations exist, but future studies should keep exploring mechanisms of change. For example, in a previous study we found depressive symptoms mediated the longitudinal link between insomnia symptoms and later NSSI, while we found no evidence that impulsivity mediated this link^30^. Finally, it is possible that the associations found in this study were attenuated by the attrition driven by insomnia symptoms as well as underreporting due to stigma surrounding self-harming behaviors, despite our efforts to reassure participants about confidentiality (e.g., ensuring no teachers were present). Nevertheless, the results of this study are based on a low-risk sample, and future studies should also address these temporal associations in clinical samples.

Longitudinal studies have not only shown that adolescents who sleep poorly harm themselves, but that they are also at higher risk for attempting suicide later on^34^. Therefore, understanding which trajectories of sleep problems are associated with NSSI is important for identifying which adolescents are in need of targeted prevention and/or intervention. Our results clearly showed that adolescents who reported persistent sleep problems (both insomnia symptoms and short sleep) over time also reported more self-injurious behaviors. Importantly, adolescents whose insomnia symptoms improved over time reported lower NSSI frequency compared to adolescents reporting persistent insomnia symptoms, which suggests that interventions improving sleep may effectively reduce the risk for NSSI in this population. Finally, measuring NSSI in adolescents with elevated insomnia symptoms may help identify a risk-group for both chronic sleep problems and persistent self-injurious behavior.

## Methods

### Design & participants

The sample for this study was drawn from a longitudinal study (The Three Cities’ Study). The data were collected in 18 public schools, during school hours, in three middle-sized cities in Sweden, including 7th grade students (age 13) in 2014 (Time 1), followed up one year later in 8th grade in 2015 (Time 2), and 9th grade (Time 3) in 2016. Among the participating 7th grade students (N = 1,457; 47.3% girls), only those with data on insomnia and sleep duration at Time 1 were included (N = 1,294, *M*_*age*_ = 13.2, *SD* = 0.42; 46.8% girls). Trained test leaders administered the surveys allowing students 90 min to complete the questionnaires. Each class received 300 Swedish crowns in recognition of participation. Before participation, active informed consent from students and passive informed consent from parents were received, to increase participation rate and to limit sampling bias^35,36^. Moreover, students were informed about confidentiality, that participation was voluntary and that they could choose to withdraw from the study at any time. A psychologist was available to support any concerns arising from answering some of the sensitive questions included in the questionnaire. The project was approved by the Regional Ethical Board in Uppsala, Sweden (No 2013/384) and the study was performed in accordance with relevant guidelines and regulations. The characteristics of the sample, procedure and data collection have been thoroughly described elsewhere^28^.

### Measures

#### Sleep duration

Weekday sleep duration was estimated from adolescents’ self-reported bedtime (“*What time do you usually go to bed on school days?*”), wake-time (“*What time do you usually wake up on school days?*”), and sleep onset latency (“*On school nights, after you go to bed, about how long does it take for you to fall asleep?*”) during the past two weeks. These items were drawn from the School Sleep Habits Survey^37^, which has shown good validity when compared to wrist actigraphy^33^. Weekday sleep duration was calculated as the interval between bed- and wake-time, minus sleep onset latency.

#### Insomnia

We used the 7-item Insomnia Severity Index (ISI) to measure symptoms of insomnia among adolescents^38^. Items cover difficulties falling asleep, staying asleep and waking up too early as well as perceived satisfaction with sleep, interference with daytime functioning and worry about sleep. The time frame in this study was changed from 2-weeks (in the original) to the last 6 months to match how somatic complaints were measured in the rest of the survey. Response option ranges from 0–4, with higher scores indicating more severe problems and a possible total score of 28. The scale has been shown to be reliable and valid^39^. In the present study, Cronbach’s alphas were 0.82 at T1, 0.84 at T2, and 0.87 at T3.

#### Non-suicidal self-injury

NSSI was measured using a shortened version of the original Deliberate Self-Harm Inventory^40^. A stem was added so that participants reported only non-suicidal injuries^41^. Adolescents responded to nine items about the extent to which they had inflicted non-lethal injuries on themselves in the last 6 months and without suicidal intent. The responses format ranged from *0 times* (0) to *more than 5 times* (6) to questions such as “*In the last 6 months have you…purposely cut your wrists, arms, or some other part of your body?”* and included the following behaviors: cutting, scratching, burning, carving, hitting/banging, sticking, biting, preventing wounds from healing. Average scores were calculated (see Table 1). Cronbach’s alphas for this measure were 0.86 at T1, 0.84 at T2, and 0.90 at T3.

### Control variables

#### Demographics

Adolescents were asked about their sex (male/female) and age. Socioeconomic status (SES) was assessed through four questions about family affluence from the Family Affluence Scale (FAS-II) which was used in the Health Behavior in School-aged Children (HBSC)^42^. Questions about adolescents’ immigrant background included place of birth and parents’ place of birth (including Sweden, Scandinavia, Europe and outside Europe). Immigrant background was defined as being born outside of Sweden or being born in Sweden with both non-Swedish parents according to Statistics Sweden^43^.

#### Depressive symptoms

Using the Center for Epidemiologic Studies–Depression Child Scale (CES-DC)^44^, adolescents were asked how often they experienced 16 symptoms within the last week. Examples of situations were: “*I was bothered by things that don’t usually bother me*”; “*I wasn’t able to feel happy, even when my family or friends tried to help me feel better*”. Answers ranged from *not at all* (0) to *often* (3). At T1, possible responses mistakenly ranged from 1 to 5 and were transformed to a range from 0 to 3 by using a proportion of maximum percentage (POMP) method ((rating/80)*60) for a total score of 60, consistent with the following waves. In our study, Cronbach’s alpha was 0.95.

### Data analysis

We used a two-step analysis^45^ to 1) examine changes in sleep duration and insomnia symptoms over time, using latent curve growth model and 2) to identify different trajectories of insomnia symptoms and sleep duration, using latent class growth analysis^28^. In the present study, we compared adolescents in each trajectory on NSSI frequency, controlling for sex, immigrant background, SES, and baseline depressive symptoms using MANCOVA.

Data for this study were analyzed in SPSS v26. We handled missing data using estimation maximization (EM) in SPSS, which performs better compared to mean imputation, listwise deletion, or pairwise deletion^46^.

### Attrition analysis

Retention rate from T1 to T3 was 82.8% in the analytic sample (*n* = 1,071). We ran a logistic regression to examine if dropout was systematic, predicted attrition at T3 (1 = attrition; 0 = retention). Predictors included demographic variables (i.e., sex, immigrant background, and SES), depressive symptoms, NSSI, and sleep variables (i.e., sleep duration and insomnia). The results showed that having an immigrant background (Wald = 9.34, *p* = 0.002, OR = 1.70, CI: 1.15–2.52), and reporting insomnia symptoms (Wald = 11.35, *p* < 0.004, OR = 1.06, CI: 1.02–1.11) were significant predictors of attrition at T3 (Nagelkerke R^2^ = 0.061).

## Data Availability

The data underlying this article cannot be shared publicly due to the agreement made with the individuals that participated in the study. The data will be shared on reasonable request to the corresponding author.

## Abbreviations

NSSI: Non-suicidal self-injury
ISI: Insomnia Severity Index
TST: Total sleep time
